# A midtrimester dynamic ultrasound and growth-deviation model for predicting preterm birth in velamentous cord insertion: development and risk stratification

**DOI:** 10.3389/fgwh.2026.1752352

**Published:** 2026-04-02

**Authors:** Xin Wang, Le Chang, Zhihui Liu, Hongjing Bao

**Affiliations:** 1 Inner Mongolia Autonomous Region Maternal and Child Health Care Hospital, Hohhot, China; 2 Hohhot First Hospital, Hohhot, China; 3 Xilingol League Central Hospital, Xilinhot, China

**Keywords:** delivery timing, dynamic ultrasonography, prediction model, risk stratification, velamentous cord insertion

## Abstract

**Objective:**

This study aims to develop a model to predict preterm birth in patients with velamentous cord insertion (VCI) during the second trimester.

**Methods:**

This retrospective cohort study enrolled 240 singleton pregnancies (135 VCI cases and 105 controls) from January 2023 to December 2024. Four prediction models were constructed using univariate analysis and LASSO regression: Model 1 (midtrimester clinical parameters), Model 2 (late-pregnancy ultrasound parameters), Model 3 (combined mid- and late-pregnancy parameters), and Model 4 (midtrimester growth deviation indices, including BPD_FL_ and Z_doppler_). Model performance was assessed using the AUC, sensitivity, specificity, and calibration. A three-tier risk stratification system was established based on predicted probabilities.

**Results:**

The preterm birth rate was significantly higher in the VCI group than in the control group (17.8% vs. 5.7%, *P* < 0.001). Model 4, relying exclusively on midtrimester data (21 ± 2 weeks), achieved an AUC of 0.801 (bootstrap AUC 0.809, 95% CI: 0.710–0.898), with 70.8% sensitivity and 79.3% specificity. While its discriminative performance was lower than that of late-pregnancy-based models (Model 2 AUC 0.972), Model 4 provided approximately 16 weeks of advance warning. Risk stratification identified low-risk (6.5%), moderate-risk (15.6%), and high-risk (46.4%) groups, each demonstrating distinct preterm birth rates.

**Conclusion:**

The midtrimester ultrasound deviation model enables early identification of high-risk VCI pregnancies, facilitating individualized delivery timing decisions while minimizing unnecessary interventions.

## 1 Introduction

Velamentous cord insertion (VCI) is an umbilical cord attachment anomaly in which the cord inserts into the fetal membranes, with umbilical vessels coursing between the amnion and chorion before entering the placenta. Its incidence is approximately 7.8% in singleton pregnancies and up to 16.9% in twin pregnancies (1, 2). Due to its unique anatomical structure, VCI predisposes the umbilical vessels to compression and rupture complications due to the lack of Wharton's jelly protection, increasing risks of fetal growth restriction, preterm birth, and fetal distress (3).

Currently, the optimal delivery timing for pregnancies complicated by VCI remains controversial. Premature pregnancy termination may cause iatrogenic preterm birth and associated complications, while excessive expectant management may delay optimal intervention and increase the risk of adverse events in late pregnancy (4). In the general obstetric population, several preterm birth risk prediction approaches and decision-support tools have been developed, commonly integrating maternal history with midtrimester screening markers to guide surveillance and prevention strategies. Current guideline-based frameworks emphasize ultrasound-based predictors, such as transvaginal cervical length assessment (with or without adjunct biomarkers in selected settings), as central components of preterm birth prediction and risk stratification (5). Existing studies focus primarily on VCI diagnosis and its associated pregnancy outcomes, lacking evidence-based models to guide delivery timing (6, 7). Traditional VCI management relies mainly on single ultrasound examinations and clinical experience, and lacks dynamic monitoring strategies and quantitative risk assessment tools (8).

Recent advances in ultrasound technology have shown that umbilical artery flow parameters [including systolic/diastolic ratio (S/D), resistance index (RI), and pulsatility index (PI)] are valuable for assessing placental function (9). However, single-timepoint imaging parameters may not comprehensively reflect dynamic changes in placental function. Dynamic monitoring of imaging parameter trends may more accurately assess the functional status of the fetoplacental unit than individual examinations (10). A progressive increase in blood flow resistance suggests deterioration of placental function, while slowed fetal growth reflects cumulative effects of chronic hypoxia (11). Integrating these dynamic change indicators into prediction models may improve the accuracy of risk assessments.

Notably, accurate prediction of preterm birth risk during midtrimester (21 ± 2 weeks) would have greater clinical significance. Compared to short-term predelivery prediction, midtrimester warning provides clinicians with more adequate intervention windows, facilitating individualized pregnancy management strategies, appropriate surveillance frequency arrangements, timely preventive measures, and sufficient time for patients and families to prepare psychologically. This early warning approach optimizes medical resource allocation and may improve maternal–fetal outcomes through timely intervention, representing a shift from “passive response” to “active prevention” in clinical philosophy.

Based on this background, we aimed to analyze dynamic changes in ultrasound imaging parameters among VCI patients, combined with clinical characteristics, to construct predictive models for preterm birth risk and decision systems for delivery timing, providing individualized, precise management approaches for clinical practice while maximizing improvements in clinical outcomes of VCI patients under maternal–fetal safety assurance.

## 2 Methods

### 2.1 Inclusion and exclusion criteria

The inclusion criteria were as follows: (1) singleton pregnancy with ≥28 weeks of gestation; (2) complete routine prenatal care and delivery at our hospital; (3) availability of complete ultrasound examination records from the midtrimester (21 ± 2 weeks) and the late trimester (within 1 week before delivery); and (4) availability of complete clinical data. Pregnancies delivered before 28 weeks of gestation were excluded to ensure that the study focused on a clinically actionable preterm window, during which antenatal interventions and delivery timing decisions could meaningfully modify maternal and neonatal outcomes. The exclusion criteria were as follows: (1) fetal chromosomal abnormalities or severe structural malformations; (2) concurrent placenta previa; (3) severe maternal comorbidities (including severe heart disease or renal insufficiency); and (4) missing ultrasound or clinical data that prevented calculation of dynamic parameters.

### 2.2 General information

This single-center retrospective cohort study followed internationally recognized standards for reporting prediction model research. We included 240 singleton pregnancies, all registered and delivered at the Department of Obstetrics, Inner Mongolia Autonomous Region Maternal and Child Health Care Hospital, from January 2023 to December 2024. VCI was diagnosed when a prenatal ultrasound suggested velamentous cord attachment confirmed by postpartum pathological examination. Vasa previa was defined as fetal vessels crossing or approaching the cervical os ≤2 cm (12). Patient age ranged from 22 to 44 years (30.83 ± 4.16 years). The ethnic distribution was as follows: Han (204, 85%), Mongolian (28, 11.67%), Manchu (5, 2.08%), Hui (2, 0.83%), and Bai (1, 0.42%). Gravidity ranged from 1 to 7; parity ranged from 0 to 3; abortions ranged from 0 to 4. The VCI group included 135 cases, and the control group included 105 cases. This study was approved by the hospital ethics committee [approval number: (2025) Ethics Review (011-1)]. The requirement for informed consent was waived due to the retrospective nature of the study.

### 2.3 Ultrasound examination

We used GE Voluson E10 and Philips EPIQ 7 color Doppler ultrasound systems equipped with 3–8 MHz probes. Patients were positioned supine, with probes placed on the abdominal wall to perform transverse, longitudinal, and oblique multidirectional scans to observe fetal anatomy and attachment; standardized fetal growth measurements were obtained, followed by color and spectral Doppler ultrasound for blood flow assessment and umbilical vessel evaluation at the placental insertion site and their relationship to the cervical os. Image acquisition was standardized using fixed scanning parameters (frequency, gain, depth) to reduce interdevice variability. Two senior sonographers with ≥5 years of diagnostic experience independently completed measurements in a blinded fashion.

### 2.4 Observation indicators and criteria

Actual delivery time served as the primary endpoint, with preterm birth defined as delivery at ≥28 weeks but <37 weeks of gestation. Delivery gestational age was further categorized as preterm (≥28 to <37 weeks), early term (37–39 weeks), and full term (≥39 weeks). The lower limit of 28 weeks follows current recommendations from the 2024 Clinical Prevention and Treatment Guidelines for Preterm Birth by the Obstetrics Group of the Chinese Medical Association (13).

Pregnancy complications included diabetes (diagnosed per International Association of Diabetes and Pregnancy Study Group criteria), hypothyroidism (TSH >2.5 mIU/L), hypertensive disorders (systolic BP ≥140 mmHg and/or diastolic BP ≥90 mmHg), hyperlipidemia, uterine fibroids, ovarian cysts, cord entanglement, uterine scarring, vaginitis, premature rupture of membranes, and abnormal pregnancy weight changes.

Imaging indicators were collected in two phases: early ultrasound at 21 ± 2 weeks and late ultrasound within 1 week before delivery. Parameters included fetal biometric measurements [biparietal diameter (BPD), femur length (FL), abdominal circumference (AC)], maximum amniotic fluid depth, umbilical artery S/D, RI, PI, and placental position and morphology. In the late phase, estimated fetal weight (EFW), fetal heart rate, and placental functional grading (0–III) were also measured.

Traditional dynamic change indicators (requiring both mid- and late-trimester data) were calculated as follows: (late value−early value)/examination interval (weeks): the calculated parameters included ΔBPD_std and ΔFL_std (mm/week), umbilical artery ΔS/D, ΔRI (0.55−late RI), ΔPI (0.78−late PI), and Δamniotic fluid depth (mm/week), based on longitudinal study results by Acharya et al. (10). These were used only for exploratory analyses and for Model 3 construction.

### 2.5 Prediction model construction and validation

#### 2.5.1 Variable screening and model construction strategy

For the construction of Models 1–3, we employed a three-step approach incorporating LASSO regularization. First, candidate variables (demographics, complications, ultrasound parameters, and traditional dynamic indicators including ΔBPD_std) were screened using univariate logistic regression, with a threshold of *P* ≤ 0.10. Second, LASSO regression was applied to address multicollinearity and select variables. Because ΔBPD_std demonstrated a non-linear association with preterm birth risk, it was categorized into quartiles (Q1–Q4). Third, variables with non-zero LASSO coefficients were entered into a multivariate logistic regression model.

For the construction of Model 4, the revised Model 4 adopted a theory-driven, prespecified architecture to ensure the requirement of only midtrimester data and to enhance physiological interpretability. Based on clinical rationale and correlation analysis (PI and RI *r* > 0.7), we directly specified four variables without LASSO screening: maternal age, presence of vasa previa, BPD-FL (continuous), and *Z* (continuous). These variables were simultaneously entered into a multivariate logistic regression model, with odds ratios (ORs) and 95% confidence intervals calculated for each predictor.

#### 2.5.2 Four prediction models

Based on correlation analysis and clinical operability, we constructed four prediction models, each designed to have distinct clinical roles in different VCI scenarios.

Model 1 (midtrimester risk assessment) and Model 4 were designed as prospective early-warning models, relying exclusively on information available at the midtrimester (21 ± 2 weeks) to support early risk stratification and clinical surveillance planning. Model 1 included selected baseline variables (e.g., age, advanced maternal age status, total number of comorbidities, 21-week BPD, FL, and umbilical artery RI) to provide a baseline midtrimester risk assessment. In contrast, Model 2 (late comprehensive assessment) and Model 3 (mid + late combined) incorporated ultrasound parameters obtained within 1 week before delivery. These models were constructed as near-delivery benchmark models rather than early prediction tools. Specifically, Model 2 included predelivery ultrasound parameters (BPD, FL, AC, S/D, and EFW) to represent the theoretical upper bound of predictive performance achievable when late-pregnancy information is available, while Model 3 integrated both mid- and late-pregnancy static ultrasound parameters to evaluate the combined prediction value of cross-temporal integration.

To enable pure midtrimester prediction for the Model 4, we introduced a population-based growth deviation metric that requires only 21 ± 2 weeks of data. The calculation process is described as follows: (1) Reference growth curve construction: In the control group (non-VCI), we established normal growth curves for BPD, FL, PI, and RI using quadratic polynomial regression against exact gestational age at the midtrimester (130–200 days). These curves represent the expected trajectory for a healthy population at each gestational age. (2) *Z*-score deviation calculation: For each VCI patient, we calculated standardized deviations (*Z*-scores) representing the magnitude of deviation from these age-specific norms: *Z* = (observed value−predicted value for the same GA)/residual SD. This approach captures growth and placental function patterns during the midtrimester without requiring late-pregnancy measurements. (3) Composite indices: To address multicollinearity between PI and RI (*r* > 0.7), we constructed a composite Doppler deviation magnitude score: Z_Doppler_ = √(PI_z_^2^ + RI_z_^2^)/√2. This metric represents the Euclidean distance between the combined PI and RI deviations and gestational-age-specific reference values. Because the *Z*-scores are squared, Z_Doppler_ is non-negative and directionless—it quantifies the magnitude of Doppler deviation regardless of whether PI/RI are above or below expected values. In addition, we calculated a head-to-femur growth discordance index: BPD-FL_diff_ = BPD_z_−FL_z_, capturing the characteristic brain-sparing, asymmetric growth pattern observed in cases of placental insufficiency.

#### 2.5.3 Model performance evaluation

Discrimination was assessed using the area under the receiver operating characteristic (ROC) curve (AUC) with 95% confidence intervals. Optimal cutoff values were determined by the Youden index (maximum sensitivity + specificity−1). Calibration was assessed using the Hosmer–Lemeshow goodness-of-fit test (*χ*^2^ test, *g* = 10 groups), which evaluates the concordance between predicted probabilities and actual outcomes, with *P* > 0.05 indicating good calibration. Internal validation was performed using 1,000-fold bootstrap resampling to assess model stability, calculate bias-corrected AUC estimates, and obtain 95% confidence intervals (percentile method). Clinical utility was evaluated using decision curve analysis (DCA), which evaluates the net clinical benefit across different risk thresholds. Net benefit was calculated using the following formula: NB = (TP/*n*)−(FP/*n*) × [Pt/(1−Pt)], where Pt = risk threshold, TP = true positives, FP = false positives, and *n* = total sample size. The net benefit of Model 4 was compared with extreme strategies of “treat all” and “treat none” across thresholds ranging from 5% to 50%, quantifying the proportion of avoidable excessive interventions.

### 2.6 Construction and validation of a preterm birth risk stratification system

#### 2.6.1 Survival analysis of cumulative risk differences

The Kaplan–Meier method was used to estimate cumulative preterm birth incidence in the VCI and control groups, with the log-rank test comparing differences in cumulative risk curves. Cox proportional hazards regression was used to calculate hazard ratios (HRs) and 95% confidence intervals, quantifying the independent effects of VCI on preterm birth risk. Because term delivery prevents the occurrence of preterm birth (a competing event), Fine–Gray competing risk models were also employed. Event coding was defined as follows: 0 = censored (follow-up), 1 = preterm birth (event of interest), and 2 = term delivery (competing event). The Gray test was used to compare differences in cumulative incidence under the competing risk framework. Cumulative preterm birth rates at key time points (32, 34, 36, and 37 weeks) were estimated using the cumulative incidence function (CIF), with confidence intervals calculated using the Greenwood formula.

#### 2.6.2 Three-tier risk stratification system

Based on the output of Model 4, we constructed a three-tier risk stratification system using individual predicted probabilities. Risk thresholds were set based on clinical significance, with values of 0.1 and 0.3, categorizing VCI patients into low-risk (<0.1), moderate-risk (0.1–0.3), and high-risk (>0.3) groups. The following threshold settings were considered: a statistically significant increasing trend in preterm birth rates across risk tiers; a preterm birth rate in the low-risk group approaching that of the control population; and a high-risk group with a preterm birth probability exceeding 45% in ultrahigh-risk populations.

#### 2.6.3 Statistical validation of the stratification system

The effectiveness of the risk stratification system was validated using the Cochran–Armitage linear trend test, which verifies monotonically increasing preterm birth rates across risk tiers (one-sided test, *P* < 0.05 for significant trend), Spearman rank correlation, which quantifies the correlation between the predicted probability and actual preterm outcomes (*ρ* > 0.6 indicating strong correlation), a chi-square test, which compares the differences in overall preterm birth rates among three risk tiers, and the Fisher exact test, which compares the preterm birth rate equivalence between the low-risk group and control group (*P* > 0.05 indicating no significant difference, supporting reasonable stratification). The 95% confidence intervals for risk-tier preterm birth rates were calculated using the Clopper–Pearson exact method, thereby avoiding the bias associated with normal approximation in small samples or extreme proportions.

### 2.7 Individualized clinical management strategies

Based on the risk stratification results, we developed individualized management strategies. The low-risk group continued routine prenatal care and underwent expectant management with planned term natural delivery. The moderate-risk group received enhanced monitoring with planned delivery at 37–38 weeks of gestation. Within the high-risk group, a risk-stratified management strategy was adopted: patients with higher predicted probabilities received multidisciplinary intensive monitoring with planned delivery before 37 weeks, while those with relatively lower but still elevated probabilities received enhanced monitoring with planned delivery at 37–38 weeks. Decision curve analysis validated the clinical net benefit of this stratified management strategy relative to the “treat all” and “treat none” extreme approaches across 5%–50% thresholds.

### 2.8 Statistical methods

Normality of continuous data was tested using the Shapiro–Wilk test. Normally distributed data were presented as mean ± standard deviation and compared between groups using independent *t*-tests; non-normally distributed data were presented as median (P25, P75) and compared using the Mann–Whitney *U*-test. Categorical variables were presented as counts (%)and compared using the chi-square test or Fisher’s exact test. Categorical variables with multiple categories were analyzed using the chi-square test for overall group differences. Correlation analysis was performed using Pearson or Spearman correlation coefficients, depending on data distribution. Interobserver agreement for ultrasound parameters was assessed using the intraclass correlation coefficient (ICC): ICC<0.50, poor agreement; 0.50 ≤ ICC<0.75, moderate agreement; 0.75 ≤ ICC<0.90, good agreement; ICC≥0.90, excellent agreement. All tests were two-sided, with *P* < 0.05 indicating statistical significance. Statistical analyses were performed using R software (version 4.3.1).

## 3 Results

Ultrasound workstations automatically acquired ultrasound parameters without the need for agreement testing. Placental functional grading and vasa previa diagnosis were independently assessed by two senior physicians qualified in prenatal diagnosis, with consensus reached when disagreement occurred. Agreement analysis showed excellent agreement, with kappa = 0.94 for placental functional grading and 0.91 for vasa previa.

### 3.1 VCI preterm birth risk factor analysis

Comparison of clinical characteristics between the VCI and control groups showed no significant differences in ethnic distribution, gravidity, parity, or history of abortion (all *P*’s > 0.05). For pregnancy-related indicators, gestational age was lower in the VCI group (*P* < 0.001). The VCI group also exhibited a significantly higher proportion of preterm birth and early-term births and a lower proportion of full-term births compared with the control group (*P* < 0.001). Regarding complications, the incidence of gestational hypertension was higher in the VCI group (*P* = 0.041). The VCI group also exhibited a significantly higher incidence of vasa previa (*P* = 0.001). In addition, rates of cesarean delivery, low birth weight, and composite adverse outcomes were higher in the VCI group (all *P*’s < 0.01). No significant differences were found between the two groups in terms of weight prediction deviation, total number of comorbidities, or other pregnancy-related complications (all *P*’s > 0.05) (Table 1).

**Table 1:** Basic characteristics of the study population.

Clinical features		Control group (105 cases)	VCI group (135 cases)	Statistic	*P-*value
Basic demographic characteristics	Age (Q1,Q3), years	30.0 (28.0, 32.0)	30.0 (28.0, 34.0)	*Z* = −1.42	0.156
	Nationality [*n* (%)]			*χ*^2^ = 1.36	0.937
	Bai	0 (0.00)	1 (0.74)		
	Han Chinese	89 (84.76)	115 (85.19)		
	Hui	1 (0.95)	1 (0.74)		
	Manchu	3 (2.86)	2 (1.48)		
	Mongol	12 (11.4)	16 (11.9)		
Obstetric history	Gravidity [*n* (%)]			*χ*^2^ = 4.77	0.614
	1	54 (51.43)	60 (44.44)		
	2	25 (23.81)	39 (28.89)		
	3	13 (12.38)	21 (15.56)		
	4	10 (9.52)	12 (8.89)		
	5	3 (2.86)	1 (0.74)		
	6	0 (0.00)	1 (0.74)		
	7	0 (0.00)	1 (0.74)		
	Parity [*n* (%)]			*χ*^2^ = 5.42	0.127
	0	73 (69.52)	95 (70.37)		
	1	29 (27.62)	38 (28.15)		
	2	3 (2.86)	0 (0.00)		
	3	0 (0.00)	2 (1.48)		
	Abortus [*n* (%)]			*χ*^2^ = 2.45	0.694
	0	66 (62.86)	77 (57.04)		
	1	23 (21.90)	36 (26.67)		
	2	13 (12.38)	16 (11.85)		
	3	3 (2.86)	4 (2.96)		
	4	0 (0.00)	2 (1.48)		
Pregnancy outcome indicators	Gestational age (Q1,Q3), weeks	39.7 (38.9, 40.6)	38.9 (37.5, 39.9)	*Z* = −4.38	<0.001
	Gestational age at delivery [*n* (%)]			*χ*^2^ = 16.28	<0.001
	Preterm: <259 days	6 (5.71)	24 (17.78)		
	Early-term: 259–272 days	21 (20.00)	44 (32.59)		
	Full-term: ≥273 days	78 (74.29)	67 (49.63)		
	Caesarean section rate [*n* (%)]	42 (40.0)	107 (79.3)	*χ*^2^ = 37.02	<0.001
	Low birth weight [*n* (%)]	4 (3.8%)	22 (16.3%)	*χ*^2^ = 8.28	0.004
	Estimated fetal weight deviation [*n* (%)]	2 (1.9%)	9 (6.7%)	*χ*^2^ = 2.07	0.15
	Composite adverse outcome [*n* (%)]	8 (7.6%)	32 (23.7%)	*χ*^2^ = 9.87	0.002
Maternal comorbidities	Total number of complications (Q1,Q3)	1.0( 0.0, 1.0)	1.0 (0.0, 1.0)	*Z* = −0.94	0.349
	Gestational diabetes mellitus [*n* (%)]			*χ*^2^ = 0.13	0.723
	No	92 (87.62)	115 (85.19)		
	Yes	13 (12.38)	20 (14.81)		
	Hypothyroidism during pregnancy [*n* (%)]			*χ^2^* = 0.04	0.841
	No	82 (78.10)	108 (80.00)		
	Yes	23 (21.90)	27 (20.00)		
	Uterine fibroids [*n* (%)]			*χ*^2^ = 0.26	0.608
	No	92 (87.62)	114 (84.44)		
	Yes	13 (12.38)	21 (15.56)		
	Hyperlipidaemia [*n* (%)]			*χ*^2^ = 0.03	>0.999
	No	103 (98.10)	132 (97.78)		
	Yes	2 (1.90)	3 (2.22)		
	Hypertensive disorders of pregnancy [*n* (%)]			*χ*^2^ = 4.17	0.041
	No	99 (94.29)	115 (85.19)		
	Yes	6 (5.71)	20 (14.81)		
	Ovarian cyst [*n* (%)]			*χ*^2^ = 0.00	>0.999
	No	102 (97.14)	131 (97.04)		
	Yes	3 (2.86)	4 (2.96)		
	Umbilical cord entanglement [*n* (%)]			*χ*^2^ = 0.01	0.932
	No	67 (63.81)	88 (65.19)		
	Yes	38 (36.19)	47 (34.81)		
	Weight loss [*n* (%)]			*χ*^2^ = 1.19	0.275
	No	91 (86.67)	124 (91.85)		
	Yes	14 (13.33)	1 1 (8.15)		
	Weight gain [*n* (%)]			*χ*^2^ = 2.03	0.154
	No	71 (67.62)	78 (57.78)		
	Yes	34 (32.38)	57 (42.22)		
	Uterine sca [*n* (%)]			*χ*^2^ = 0.00	0.981
	No	92 (87.62)	117 (86.67)		
	Yes	13 (12.38)	18 (13.33)		
	Vaginitis [*n* (%)]			*χ*^2^ = 0.12	0.724
	No	95 (90.48)	125 (92.59)		
	Yes	10 (9.52)	10 (7.41)		
	Prelabor rupture of membranes [*n* (%)]			*χ*^2^ = 2.49	0.115
	No	82 (78.10)	117 (86.67)		
	Yes	23 (21.90)	18 (13.33)		
	Vasa previa [*n* (%)]			*χ*^2^ = 11.50	0.001
	No	105 (100.00)	119 (88.15)		
	Yes	0 (0.00)	16 (11.85)		

*Z* = Mann–Whitney *U*-test; *χ*^2^ = chi-square test; VCI, velamentous cord insertion.

Comparison of ultrasound parameters between the VCI and control groups showed no significant differences in early ultrasound parameters at 21 ± 2 weeks (head circumference, BPD, FL, AC, lateral ventricle width, cerebellar diameter, humeral length, fetal heart rate, RI, PI, amniotic fluid depth, and S/D ratio) (all *P*’s > 0.05). Within 1 week before delivery, the VCI group demonstrated significantly smaller late-pregnancy BPD, FL, and AC compared with the control group (all *P*’s < 0.05), while no significant differences were observed in late S/D, amniotic fluid depth, and placental functional grading (*P* > 0.05). For delivery-related indicators, maternal abdominal circumference was greater in the VCI group than in the control group (*P* = 0.037). The distribution of vertex/breech presentation was similar between groups (*P* > 0.05), while the distribution of cervical ripening scores differed significantly (*P* = 0.046), with scores in the VCI group concentrated at 3–4 points and controls having a relatively higher proportion of scores ≥5 points (Table 2).

**Table 2:** Ultrasonographic characteristics of the study population.

Clinical features		Control group (105 cases)	VCI group (135 cases)	Statistic	*P-*value
Early ultrasound parameters (21 ± 2 weeks of gestation)	Early head circumference (Q1, Q3)	20.1 (19.6, 20.8)	20.0 (19.5, 20.5)	*Z* = −1.02	0.306
	Early biparietal diameter (Q1, Q3)	5.4 (5.2, 5.6)	5.4 (5.2, 5.5)	*Z* = −0.35	0.726
	Early femur length (Q1, Q3)	3.8 (3.6, 3.9)	3.8 (3.7, 3.9)	*Z* = −1.21	0.224
	Early abdominal circumference (Q1, Q3)	18.1 (17.3, 18.8)	17.8 (17.1, 18.4)	*Z* = −1.60	0.109
	Early lateral ventricle width (Q1, Q3)	0.6 (0.5, 0.7)	0.6 (0.5, 0.6)	*Z* = −0.71	0.476
	Early transcerebellar diameter (Q1, Q3)	2.4 (2.3, 2.5)	2.4 (2.3, 2.4)	*Z* = −0.56	0.572
	Early humerus length (Q1, Q3)	3.5 (3.4, 3.7)	3.5 (3.4, 3.6)	*Z* = −1.61	0.108
	Early fetal heart rate (Q1, Q3)	148.0 (143.0, 154.0)	149.0 (143.0, 153.0)	*Z* = −0.16	0.87
	Early resistance index (Q1, Q3)	0.7 (0.6, 0.7)	0.7 (0.6, 0.7)	*Z* = −0.80	0.424
	Early pulsatility index (Q1, Q3)	1.1 (1.0, 1.2)	1.1 (1.0, 1.2)	*Z* = −0.36	0.718
	Early amniotic fluid depth (Q1, Q3)	4.6 (4.3, 5.4)	4.9 (4.4, 5.2)	*Z* = −1.39	0.165
	Early S/D ratio (mean ± SD)	3.0 ± 0.5	3.0 ± 0.4	t = 1.11	0.269
Late ultrasound parameters (1 week before delivery)	Late biparietal diameter (Q1, Q3)	9.3 (9.2, 9.6)	9.3 (9.0, 9.5)	*Z* = −2.44	0.015
	Late femur length (Q1, Q3)	7.1 (6.9, 7.3)	6.9 (6.7, 7.2)	*Z* = −4.13	<0.001
	Late abdominal circumference (Q1, Q3)	34.0 (32.8, 35.1)	33.1 (31.4, 34.5)	*Z* = −3.55	<0.001
	Late S/D ratio (Q1, Q3)	2.1 (1.9, 2.4)	2.2 (1.9, 2.5)	*Z* = −0.86	0.39
	Late amniotic fluid depth (Q1, Q3)	4.8 (4.4, 5.4)	5.0 (4.4, 5.7)	*Z* = −1.54	0.124
	Placental function [*n* (%)]			*χ*^2^ = 4.64	0.068
	1	14 (13.33)	33 (24.44)		
	2	90 (85.71)	101 (74.81)		
	3	1 (0.95)	1 (0.74)		
Delivery-related indicators	Maternal abdominal circumference (Q1, Q3)	100.0 (96.0, 106.0)	103.0 (98.0, 106.0)	*Z* = −2.09	0.037
	Cephalic presentation [*n* (%)]			*χ*^2^ = 2.22	0.136
	No	4 (3.81)	13 (9.63)		
	Yes	101 (96.19)	122 (90.37)		
	Breech presentation [*n* (%)]			*χ*^2^ = 2.22	0.136
	No	101 (96.19)	122 (90.37)		
	Yes	4 (3.81)	13 (9.63)		
	Cervical maturity (Bishop) score [*n* (%)]			*χ*^2^ = 14.22	0.046
	1	1 (0.95)	4 (2.96)		
	2	18 (17.14)	19 (14.07)		
	3	40 (38.10)	66 (48.89)		
	4	15 (14.29)	28 (20.74)		
	5	20 (19.05)	11 (8.15)		
	6	6 (5.71)	6 (4.44)		
	7	3 (2.86)	1 (0.74)		
	8	1 (0.95)	0 (0.00)		
	10	1 (0.95)	0 (0.00)		

*Z*, Mann–Whitney *U*-test; *χ*^2^, chi-square test; VCI, velamentous cord insertion.

Subgroup analysis within the VCI group (with/without vasa previa) showed that patients with vasa previa (16 cases) had a mean gestational age of 37.2 weeks and a cesarean delivery rate of 100%; in contrast, patients without vasa previa (119 cases) had a mean gestational age of 38.6 weeks and a cesarean delivery rate of 76.5%.

### 3.2 Construction of the preterm birth prediction model

Exploratory analyses for Models 1–3: Correlation analysis showed that maternal age was negatively correlated with gestational age (*r* = −0.155, *P* < 0.016). Among late ultrasound parameters, BPD, AC, EFW, and FL were positively correlated with gestational age (*r* = 0.685, 0.687, 0.721, and 0.167, respectively, all *P*’s < 0.001), while the traditional dynamic indicator ΔBPD_std was significantly negatively correlated with gestational age (*r* = −0.369, *P* < 0.001). Univariate logistic regression analysis of candidate variables for Models 1–3 indicated that age, BPD, FL, AC, EFW, VCI confirmation, presence of vasa previa, and ΔBPD_std were associated with preterm birth (all *P*’s < 0.05) (Table 3).

**Table 3:** Univariate analysis of preterm delivery in patients.

Clinical factors	*β*	Wald *z*	OR (95% CI)	*P-*value
Age	0.1205	2.662	1.128 (1.032–1.233)	0.008
BPD	−4.1926	−6.046	0.015 (0.004–0.059)	<0.001
FL	−6.3851	−5.954	0.002 (0.000–0.014)	<0.001
AC	−1.3062	−5.839	0.323 (0.221–0.472)	<0.001
Estimated fetal weight	−0.0029	−6.306	0.997 (0.996–0.998)	<0.001
VCI_confirmed	1.2719	2.667	3.57 (1.40–9.08)	0.008
Vasa previa	2.2172	4.045	9.182 (3.136–26.882)	<0.001
ΔBPD_std (Q2 vs. Q1)	−2.3179	−2.121	0.098 (0.011–0.872)	0.034
ΔBPD_std (Q3 vs. Q1)	−0.5792	−0.918	0.560 (0.163–1.925)	0.359
ΔBPD_std (Q4 vs. Q1)	0.3457	0.624	1.413 (0.477–4.189)	0.533

*β*, regression coefficient; Wald *z*, Wald’s test statistic; OR, odds ratio; 95% CI, 95% confidence interval; BPD, biparietal diameter; FL, femur length; AC, abdominal circumference; VCI, velamentous cord insertion; ΔBPD_std, standardized change rate of biparietal diameter (quartile grouping, with Q1 as the reference); Q1, slowest-growth group, Q2–Q4, progressively faster-growth groups.

Comparison of the four predictive models demonstrated that Model 2 (pure late assessment) achieved the highest predictive performance (AUC = 0.972, bootstrap AUC = 0.975, 95% CI: 0.945–0.997), with 83.3% sensitivity and 98.2% specificity. This result represents the theoretical upper bound of predictive accuracy achievable when late-pregnancy information is available. Model 4 (midtrimester plus deviation), which relied exclusively on midtrimester information, achieved an AUC of 0.801 (bootstrap AUC = 0.809, 95% CI: 0.710–0.898), with 70.8% sensitivity and 79.3% specificity. While its discriminative ability is lower than that of Models 2 and 3, it represents a clinically acceptable tradeoff in exchange for approximately 16 weeks of advance warning. The reduction in predictive performance from Model 2 to Model 4 (ΔAUC = 0.171) quantifies the information loss associated with the absence of late-pregnancy data, yet Model 4 maintains clinically useful discriminative ability for early risk stratification. Model 3 (mid + late combined) demonstrated intermediate performance (AUC = 0.936, bootstrap AUC = 0.940, 95% CI: 0.888–0.982), with 79.2% sensitivity and 97.3% specificity, while Model 1 (pure midtrimester assessment) showed the lowest predictive performance (AUC = 0.762, bootstrap AUC = 0.763, 95% CI: 0.651–0.861). Hosmer–Lemeshow goodness-of-fit tests indicated adequate calibration for all models (*P* = 0.525, 0.943, 0.726, and 0.445 for Models 1–4, respectively; Table 4).

**Table 4:** Comparison of performance of four models.

Model	AUC	Bootstrap AUC	95% CI	Sensitivity (%)	Specificity (%)	Accuracy (%)
Model 1 (second-trimester only)	0.76	0.76	0.651–0.861	83.30	58.60	63
Model 2 (late-third-trimester only)	0.97	0.97	0.945–0.997	83.30	98.20	95.60
Model 3 (second + late third trimester)	0.94	0.95	0.906–0.991	87.50	92.80	91.90
Model 4 (second trimester + deviation)	0.80	0.81	0.710–0.898	70.8	79.3	77.8

AUC, area under the receiver operating characteristic curve; bootstrap AUC, mean AUC derived from 1,000 bootstrap resamplings; 95% CI = 95% confidence interval. Sensitivity, specificity, and accuracy were calculated at the optimal cutoff determined by the Youden index. The Hosmer–Lemeshow (H–L) test was used to assess model calibration, with *P* > 0.05 indicating adequate goodness of fit. Model 1 represents second-trimester assessment at 21 weeks of gestation; Model 2 represents late-third-trimester assessment within 1 week before delivery; Model 3 integrates second- and late-third-trimester ultrasound parameters; Model 4 represents a second-trimester dynamic monitoring model incorporating growth deviation indicators. Model 4 was reconstructed during revision to use exclusively midtrimester parameters; the performance metrics reflect the tradeoff between early prediction and discriminative performance.

### 3.3 Construction of the preterm birth risk stratification system

The study included 135 VCI patients and 105 controls. Kaplan–Meier survival analysis showed that the cumulative incidence of preterm birth in the VCI group at 32, 34, 36, and 37 weeks was 2.2%, 5.2%, 14.8%, and 17.8%, respectively, compared with 1.0%, 1.9%, 4.8%, and 5.7% in the control group. The log-rank test showed a significant difference between the survival curves (*χ*^2^ = 7.77, *P* = 0.005). Fine -Gray competing risk analysis, with term delivery as a competing event, showed a significantly higher cumulative incidence of preterm birth in the VCI group (Gray's test *χ*2 = 7.81, *P* = 0.005) (Table 5).

**Table 5:** Cumulative risk of premature delivery between the VIC group and the control group.

Gestational age (weeks)	Group	Numbers at risk	No. of events	Cumulative preterm birth rate (%)	95% CI
32	Control	104	1	1.00	0%–2.8%
32	VCI	133	3	2.20	0%–4.7%
34	Control	103	1	1.90	0%–4.5%
34	VCI	130	4	5.20	1.4%–8.9%
36	Control	101	3	4.80	0.6%–8.7%
36	VCI	117	13	14.80	8.6%–20.6%
37	Control	99	1	5.70	1.2%–10.1%
37	VCI	111	4	17.80	11.1%–24%

Log-rank *χ*^2^ = 7.77 (*P* = 0.005); Gray's test *χ*^2^ = 7.81 (*P* = 0.005).

Using predicted probabilities generated by Model 4, 135 VCI patients were stratified into three risk tiers: low risk (62 cases, 45.9%), moderate risk (45 cases, 33.3%), and high risk (28 cases, 20.7%). Actual preterm birth rates were as follows: low risk, 6.5% (4/62 cases, 95% CI: 1.8%–15.7%); moderate risk, 15.6% (7/45 cases, 95% CI: 6.5%–29.5%); and high risk, 46.4% (13/28 cases, 95% CI: 27.5%–66.1%). Cochran–Armitage linear trend test indicated a significant increasing trend in preterm birth rates across risk tiers (*χ*^2^ = 69.98, *P* < 0.001). Spearman rank correlation revealed a significant positive correlation between predicted probabilities and observed preterm birth rates (*ρ* = 0.681, *P* < 0.001). The chi-square test showed significant differences in preterm birth rates among the three risk tiers (*χ*^2^ = 73.74, df = 2, *P* < 0.001). Fisher’s exact test showed no significant difference between the preterm birth rate in the low-risk group (6.5%) and that in the control group (5.7%) (OR = 1.14, 95% CI: 0.31–4.20, *P* = 0.846) (Table 6).

**Table 6:** Performance of the three-level risk stratification system based on Model 4.

Risk stratum	Predicted probability range (%)	Patients (*n*)	Proportion (%)	Preterm births (*n*)	Preterm birth rate (%)	95% CI
Low risk	<10	62	45.9	4	6.5	1.8%–15.7%
Intermediate risk	10–30	45	33.3	7	15.6	6.5%–29.5%
High risk	>30	28	20.7	13	46.4	27.5%–66.1%
Total	–	135	100	24	17.80	–

Cochran–Armitage trend test *χ*^2^ = 69.98 (*P* < 0.001); Spearman rank correlation *ρ* = 0.681 (*P* < 0.001); *χ*^2^ test *χ*^2^ = 73.74 (df = 2, *P* < 0.001); low-risk vs. control Fisher's exact OR = 1.14 (95% CI: 0.31–4.20, *P* = 0.846).

Decision curve analysis compared the clinical net benefit of the Model 4-based stratification strategy with the treat-all and treat-none strategies across 5%–50% risk thresholds. At a 5% risk threshold, Model 4 achieved a maximum net benefit of 0.1598 compared with 0.1345 for the treat-all strategy, with a difference of 0.0253. At this threshold, Model 4 recommended intervention for 37% of patients. At a 15% risk threshold, Model 4 demonstrated a net benefit of 0.1373 compared with 0.0327 for the treat-all strategy, with a difference of 0.1046. At this threshold, Model 4 recommended intervention for 25.9% of patients, avoiding intervention in 74.1% of cases (Table 7).

**Table 7:** Net benefit comparison between model 4 and the treat-all strategy across different risk thresholds.

Risk threshold (%)	Net benefit (Model 4)	Net benefit (treat-all)	Difference in net benefit	Intervention proportion recommended by Model 4 (%)	Proportion of interventions avoided (%)
5	0.1598	0.1345	0.0253	37.00	63.00
10	0.1473	0.0864	0.0609	–	–
15	0.1373	0.0327	0.1046	25.90	74.10
20	0.1407	−0.0278	0.1685	–	–
25	0.1235	−0.0963	0.2198	–	–
30	0.1217	−0.1746	0.2963	–	–
35	0.1248	−0.265	0.3897	–	–
40	0.1259	−0.3704	0.4963	–	–
45	0.1226	−0.4949	0.6175	–	–
50	0.1037	−0.6444	0.7481	–	–

Difference in net benefit = net benefit (Model 4) – net benefit (treat-all); negative values indicate that the strategy causes more harm than benefit.

### 3.4 Typical clinical cases for Model 4 application

To demonstrate the clinical value of Model 4 in real-world decision-making, we selected two representative VCI patients for detailed analysis.

Patient 1 is a 41-year-old woman at 22 weeks of gestation (154 days) with a confirmed diagnosis of VCI. Model 4 yielded a preterm birth probability of 45.4%, classifying her as high risk. Midtrimester ultrasound demonstrated a BPD of 54.0 mm (+0.34 SD) and an FL of 36.5 mm (+0.30 SD), with minimal head-to-femur discordance (BPD_FL_diff = 0.03). The composite Doppler deviation score was Z_doppler = 0.48. Notably, the high-risk classification of this patient was predominantly driven by advanced maternal age (41 years), as fetal growth parameters remained within the normal range and Doppler indices showed only modest deviation. This patient delivered at 34 + 4 weeks, with a birth weight of 2,020 g, confirming the ability of the model to identify high-risk patients based on demographic and modest biometric deviations.

**Figure 1:**
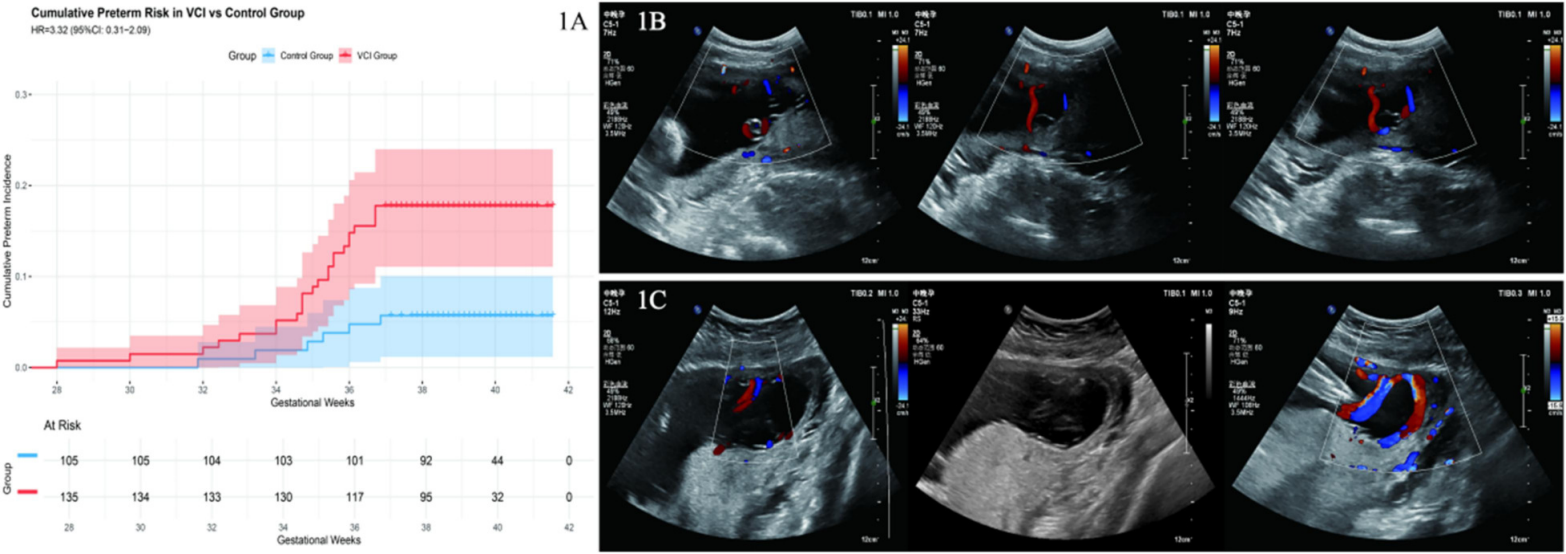
Cumulative risk of preterm birth in VCI and typical ultrasound images. **(A)** Comparison of cumulative preterm birth risk between the velamentous cord insertion (VCI) group and the control group. Kaplan–Meier curves show that the VCI group (red line) had a significantly higher cumulative risk of preterm birth than the control group (blue line); log-rank *χ*^2^ = 7.77, *P* = 0.005. Hazard ratio = 3.32 (95% CI: 1.36–8.12). Shaded areas represent 95% confidence intervals; numbers at risk at each timepoint is shown below the plot. **(B)** Ultrasound image of a high-risk VCI case (Patient 1)—a 41-year-old woman at 22 weeks of gestation. Color Doppler shows umbilical vessels (red/blue flow signals) coursing within the fetal membranes without Wharton's jelly protection. Model 4 predicted a 45.4% probability of preterm birth based on advanced maternal age (41 years), minimal growth discordance (BPD_FL_diff = 0.03), and modest Doppler deviation (Z_Doppler = 0.48); delivery occurred at 34 + 4 weeks with a birth weight of 2,020 g. **(C)** Ultrasound image of a moderate-risk VCI case (Patient 2)—a 39-year-old woman at 23 weeks of gestation. Ultrasound shows umbilical vessels within the membranes with clear flow signals. Model 4 predicted a 28.0% probability of preterm birth based on significant growth discordance (BPD_FL_diff = 1.55) and elevated Doppler deviation (Z_Dopple*r* = 1.29), counterbalanced by younger age relative to high-risk threshold; delivery occurred at 39 + 1 weeks with a birth weight of 2,030 g, indicating fetal growth restriction despite near-term gestational age.

Patient 2 is a 39-year-old woman at 23 weeks of gestation (161 days) with a confirmed diagnosis of VCI. Model 4 indicated a preterm birth probability of 28.0%, classifying her as moderate risk. Growth deviation analysis revealed a marked asymmetric growth pattern, with a BPD of 58.9 mm (+2.64 SD), an FL of 38.3 mm (+1.09 SD), and significant head-to-femur discordance (BPD_FL_diff = 1.55), consistent with a brain-sparing pattern of redistribution. The composite Doppler deviation score was elevated (Z_doppler = 1.29). Despite substantial biometric and Doppler deviations suggestive of placental insufficiency, the absence of advanced maternal age (relative to Patient 1) and vasa previa led to a moderate-risk classification. This patient delivered at 39 + 1 weeks; however, the neonate exhibited growth restriction, with a birth weight of 2,030 g, underscoring that moderate-risk classification does not preclude adverse outcomes and may warrant enhanced surveillance (Figure 1).

These cases illustrate how Model 4 integrates multiple risk dimensions—demographic, biometric, and Doppler deviations—to generate individualized risk profiles. Patient 1 exemplifies a high-risk classification driven by non-modifiable factors (age), while Patient 2 demonstrates a moderate-risk classification despite significant growth perturbations, highlighting the capacity of the model for nuanced risk stratification.

## 4 Discussion

This study successfully constructed preterm birth prediction models based on midtrimester ultrasound deviation metrics for VCI patients and established a three-tier risk stratification system, providing quantitative evidence for individualized decision-making regarding delivery timing in this special population. We confirmed a significantly increased risk of preterm birth in VCI patients (17.8% vs. 5.7%, *P* < 0.001), consistent with previous studies, further establishing VCI as an independent risk factor for preterm birth (1).

The pathophysiological basis of VCI lies in the absence of Wharton's jelly protection around the umbilical vessels, making them susceptible to compression and functional impairment as pregnancy progresses. Dynamic monitoring captures this progressive change process, with deviations in umbilical artery PI and RI from gestational-age-specific norms reflecting changes in placental vascular resistance, while midtrimester BPD and FL *Z*-scores (BPD_Z, FL_Z) capture early growth perturbations relative to population-based reference values (14, 15). Beyond hemodynamic alterations, accumulating developmental and molecular evidence indicates that abnormal placental cord insertion reflects earlier disturbances in placental morphogenesis and fetoplacental unit organization. Recent molecular analyses have demonstrated that impaired villous branching, altered trophoblast differentiation, and dysregulated angiogenic signaling can precede clinically detectable placental dysfunction, ultimately constraining placental transport capacity and fetal growth potential (16). In parallel, longitudinal imaging studies have shown that abnormal cord insertion sites are not static findings but may represent the downstream consequence of early implantation-related deviations, with limited capacity for compensatory migration during placental expansion (17). These developmental constraints provide a biological explanation for why subtle deviations in fetal biometric growth trajectories may appear before overt Doppler deterioration or clinical compromise. In this study, the head-to-femur growth discordance index (BPD_FL_diff) served as the primary predictive metric, capturing the characteristic brain-sparing pattern of asymmetric growth. The composite Doppler *Z*-score [Z_dopple_r = sqrt(PI_z_^2^ + RI_z_^2^)/sqrt(2)] complemented this by quantifying the magnitude of Doppler parameter deviations from gestational-age-specific norms, without implying directional placental function assessment. Together, these midtrimester deviation metrics provide essential evidence for early identification of high-risk patients and more sensitively reflect pathophysiological changes than single-timepoint measurements.

In this study, vasa previa, a severe complication of VCI, was associated with a markedly increased risk of preterm birth (OR = 9.18, 95% CI: 3.14–26.88), consistent with related research results (8). The 2024 published international expert consensus recommends more aggressive management strategies for patients with vasa previa, including elective cesarean delivery at 35–37 weeks, inpatient monitoring from 30–34 weeks, and fetal lung maturation between 28 and 32 weeks (18). Our results support these recommendations and further confirm that patients with vasa previa should be managed within the highest-risk group.

The core innovation of this study lies in introducing dynamic ultrasound parameters and the concepts of “growth deviation” for preterm birth prediction. Compared with traditional static parameters, dynamic monitoring models demonstrated superior predictive performance. Among the four constructed models, the late-assessment model (Model 2) achieved optimal predictive performance (AUC = 0.972), representing the theoretical upper bound achievable when late-pregnancy information is available. Model 4, relying exclusively on mid-trimester data and using four core variables (maternal age, presence of vasa previa, Z_doppler_, and BPD FL_diff_), achieved an AUC of 0.801 (bootstrap AUC = 0.809, 95% CI: 0.710–0.898). While its discriminative performance was lower than that of Models 2 and 3, this represents a clinically acceptable tradeoff in exchange for approximately 16 weeks of advance warning. The reduction in predictive performance from Model 2 to Model 4 (ΔAUC = 0.171) quantifies the information loss associated with the absence of late-pregnancy data, yet Model 4 maintains clinically useful discriminative ability for early risk stratification.

Recent developments in the field of preterm birth prediction have explored various methodological approaches. Models integrating biochemical markers have shown potential in some studies (19); however, such approaches require additional laboratory resources and cost investment, which may limit their clinical implementation. Machine learning algorithms have also demonstrated technical advantages in predicting preterm birth (20), but issues related to model complexity and interpretability may limit their adoption in primary care settings. Comparatively, our dynamic parameter-based models rely on routine ultrasound examinations and offer greater clinical operability and practicality, making them more suitable for implementation across different levels of medical institutions.

The three-tier risk stratification system based on predictive models has substantial clinical application value. The low-risk group demonstrated a preterm birth rate of only 6.5%, which was not significantly different from that of the control group, supporting the safety of expectant management with planned term delivery and aligning with recommendations from the American College of Obstetricians and Gynecologists aimed at reducing iatrogenic preterm birth (21). For the moderate-risk group, planned delivery at 37–38 weeks may reduce late-pregnancy risks while avoiding the complications of more preterm delivery. The high-risk group exhibited a preterm birth rate of 46.4%, requiring close monitoring and individualized management, especially for patients with concurrent vasa previa.

Decision curve analysis showed that this stratification strategy could avoid 74.1% of unnecessary interventions at a 15% risk threshold with good clinical net benefit. This stratified management strategy not only improves the efficiency of medical resource utilization but also provides clear decision-making evidence for clinicians. Particularly noteworthy, the midtrimester deviation model achieves early warning at 21 ± 2 weeks, approximately 16 weeks earlier than traditional predelivery prediction models, providing an adequate intervention window for clinical intervention.

The main innovations of this study include: (1) the introduction of midtrimester growth deviation *Z*-scores (BPD_Z, FL_Z, PI_Z, RI_Z) derived from population-based reference curves fitted to gestational age, enabling detection of early growth and hemodynamic perturbations at 21 ± 2 weeks before overt clinical compromise becomes evident; (2) the construction of a composite Doppler *Z*-score (Z_dopple_r) that integrates PI and RI information to address multicollinearity while preserving physiological interpretability; (3) the development of a head-to-femur growth discordance index (BPD FL_diff_) that captures the characteristic brain–sparing pattern of asymmetric growth observed in placental insufficiency; and (4) the construction of a streamlined four-variable model that achieves clinically useful early risk prediction while minimizing the risk of overfitting. Bootstrap validation results showed good model stability and generalization, establishing a foundation for clinical implementation.

Based on the study results, we recommend screening all pregnancies for cord insertion sites at 20–24 weeks, conducting dynamic monitoring every 2–4 weeks for VCI patients, implementing individualized management based on prediction models, and providing joint obstetric–neonatal management for high-risk patients. This evidence-based management strategy may improve pregnancy outcomes in VCI patients while reducing unnecessary medical interventions and maintaining maternal–fetal safety. For patients classified as high risk, structured prenatal consultation involving diverse maternal and pediatric subspecialists may further optimize decision-making and long-term outcomes. Interdisciplinary fetal–neonatal neurology training has been shown to facilitate integrated interpretation of fetal surveillance data, improve communication with prospective parents, and support developmentally informed perinatal management strategies across the lifespan (22). Such collaborative frameworks emphasize the role of specialized expertise in translating prenatal findings into individualized counseling and care planning. In addition, the incorporation of early pregnancy biochemical markers may complement imaging-based risk stratification. First-trimester biomarkers associated with uteroplacental function, such as pregnancy-associated plasma protein A (PAPP-A), have demonstrated predictive value for subsequent preterm delivery and placenta-mediated adverse outcomes (23). Together, interdisciplinary consultation and selective integration of biochemical indicators may enhance early risk recognition and refine individualized management pathways for pregnancies complicated by VCI.

However, the feasibility and impact of these recommendations are likely to vary across different levels of maternal care and health-system resources. Risk-appropriate care frameworks emphasize that access to specialized personnel, diagnostic capabilities (including sonography), and referral pathways differs substantially by facility level, which may influence the timely identification and monitoring of cord/placental anomalies, as well as the effective implementation of risk-stratified management strategies (24). In addition, an intersectionality-informed perspective is important when considering translation and equity. Multiple, overlapping factors—such as race/ethnicity, socioeconomic position, geographic distance to care, and gender-related social determinants—can jointly shape access to high-quality prenatal diagnostics and subspecialty consultation, thereby affecting real-world performance and uptake of prediction-guided pathways (25). These considerations are particularly relevant in settings where antenatal care is primarily delivered by nurse practitioners or midwives, with limited access to specialized ultrasound support. Evidence on obstetric ultrasound access and task-sharing initiatives suggests that limited availability of sonography can constrain early anomaly detection, while structured training and referral/telemedicine models may help mitigate this gap (26).

Beyond perinatal outcomes, the clinical significance of preterm birth should be interpreted within a life-course framework. The developmental origins of health and disease (DOHaD) paradigm highlights that adverse intrauterine exposures and early birth can influence health trajectories, with some consequences becoming apparent only later in childhood, adolescence, or adulthood (27). Consistent with this perspective, accumulating evidence indicates that individuals born preterm may face elevated long-term risks across multiple domains (neurodevelopmental, respiratory, and cardiometabolic), underscoring the importance of long-term follow-up beyond neonatal morbidity and mortality (28). Accordingly, future research should incorporate longer follow-up and life-course study designs to evaluate whether prediction-guided management strategies translate into sustained benefits for both women and offspring.

This study has certain limitations. First, its single-center retrospective design may limit generalizability and introduce selection bias, underscoring the need for external validation in independent, multicenter cohorts. In addition, extremely preterm births (<28 weeks) were not included, as these cases represent a distinct periviable clinical context with different management priorities and were limited in number within the present cohort; future multicenter or registry-based studies may further explore prediction models across earlier gestational ages. Moreover, the growth deviation *Z*-scores were calculated using cross-sectional, population-based reference curves fitted to gestational age rather than individualized expected growth trajectories, which introduces a degree of simplification and may not fully account for interindividual differences in growth potential; however, this approach enables early risk stratification at the midtrimester visit without the need for multiple prior measurements. Future studies incorporating repeated-measures designs or mixed-effects models could provide more personalized references for growth velocity. Second, the relatively limited sample size—particularly the small number of vasa previa cases—may affect model stability and performance across diverse populations. Although missing data were minimized by excluding cases without complete key ultrasound measurements, this complete-case approach may further contribute to selection bias. Finally, this study did not incorporate emerging predictors such as biochemical or molecular markers; future prospective studies integrating imaging, biochemical, and multiomics data are warranted to further improve predictive accuracy and clinical applicability.

## 5 Conclusion

This study successfully constructed delivery timing prediction models for VCI based on dynamic ultrasound imaging parameters, highlighting the important role of dynamic monitoring in risk assessment. Midtrimester growth deviation metrics (BPD FL_diff_ and Z_dopple_r) served as core dynamic indicators and provided reliable quantitative tools for preterm birth prediction. The established three-tier risk stratification system effectively guides clinical decision-making. Clinical application of this model may improve VCI patient pregnancy outcomes while reducing unnecessary medical interventions. Future multicenter prospective studies are needed to further validate and optimize this model.

## Data Availability

Due to patient privacy protections, individual-level data cannot be publicly shared. Aggregated data supporting the findings are available from the corresponding author upon reasonable request and with ethics committee approval.
